# In Situ Formation of Laser-Cladded Layer on Thin-Walled Tube of Aluminum Alloy in Underwater Environment

**DOI:** 10.3390/ma14164729

**Published:** 2021-08-21

**Authors:** Cheng Liu, Ning Guo, Qi Cheng, Yunlong Fu, Xin Zhang

**Affiliations:** 1 State Key Laboratory of Advanced Welding and Joining, Harbin Institute of Technology, Harbin 150001, China; liucheng@hit.edu.cn (C.L.); 13933851375@163.com (Q.C.); hitzsb@hit.edu.cn (Y.F.); zxin299@126.com (X.Z.); 2 Shandong Provincial Key Laboratory of Special Welding Technology, Harbin Institute of Technology at Weihai, Weihai 264209, China; 3 Shandong Institute of Shipbuilding Technology, Weihai 264209, China

**Keywords:** underwater laser cladding, thin-walled tube, aluminum alloy, microstructure and microhardness

## Abstract

The first study of thin-walled aluminum-alloy tubes with underwater-laser-nozzle in situ melting technology was carried out. The study mainly covered the influence of the water environment on the laser melting process, melting appearance, geometric characteristics, microstructure, regional segregation and microhardness. During the transfer of the cladding environment from air to water, the uniformity of the cladding layer became poor, but excellent metallurgical bonding was still obtained. The dilution rate (D) decreased from 0.46 to 0.33, while the shape factor (S) increased from 4.38 to 5.98. For the in-air and underwater samples, the microstructure of the melting zone (MZ) and the cladding zone (CZ) were columnar dendrites and equiaxed grains, respectively. In addition, the microstructure of the overlapping zone (OZ) was composed of columnar dendrites and equiaxed grains. The underwater average grain size was smaller than that of in-air. In addition, the water environment was beneficial for reducing the positive segregation in the columnar dendrite region. Compared with the in-air cladding sample, the precipitated phases in the OZ of the underwater cladding sample reduced. Under the combined action of grain refinement and precipitated phase reduction, the microhardness value of the underwater OZ was higher than that of the in-air OZ.

## 1. Introduction

With the overexploitation of land resources, the development and transportation of marine resources in marine engineering have developed rapidly in recent years [1,2]. However, underwater structures serve in the extreme oceanic environment, and thus they are vulnerable to corrosion failure [3]. In addition, due to the increase in water depth and the wide use of new materials, the in situ remediation of underwater components and structures began to face serious challenges [4]. There is a variety of underwater repair technologies for corroded components and structures. At present, the most widely used is underwater arc welding, but its process stability is poor, and it is still very difficult to achieve precise and high-quality underwater repair [5,6]. Therefore, high-quality, efficient and precise underwater in situ repair technologies are urgently needed. Feng et al. [7] and Guo et al. [8] pointed out that underwater laser machining technologies possess broad application prospects in the field of underwater repair. Many researchers have studied the interaction between lasers and materials in the underwater environment and have developed some precise technologies, such as underwater laser welding and underwater laser shot peening [9,10,11]. However, research on underwater laser cladding is still rare, especially on the underwater laser multiple-track cladding technology of thin-walled tubular structures.

As a key technology for the repair of stress corrosion cracks, fatigue cracks, corrosion pits and other defects of underwater structures and the surface modification of a workpiece, underwater laser cladding technology has received more and more attention in both theoretical research and engineering application fields in recent years [12]. Similar to the underwater laser welding technology, the underwater laser cladding technology could be divided into two categories: the wet method and the local dry method. In terms of underwater wet laser cladding, Cui et al. [13,14] studied the underwater wet laser cladding technology of a nickel–aluminum–bronze alloy based on the way of presetting protective coating on the base metal surface. The results show that the semi-volatile protective coating could prevent the water environment from immersing the cladding layer, reducing the shielding effect of the underwater photoplasma on the laser beam, and obtaining a continuous single cladding layer at 10 mm water depth. Furthermore, they also analyzed the effect of material composition in protective coating on the quality of the cladding layer and the comprehensive action mechanism of the laser, water and cladding layer. Jin et al. [15] adopted the protective-material-assisted method and obtained an underwater wet laser cladding layer of 316L stainless steel at a depth of 15 mm. The cladding layer was uniform and continuous without pores and cracks. By adding CaF_2_ to the protective material, the corrosion resistance and passivation ability of coating were improved. Due to the water environment and the absorption, refraction and scattering of the laser beam by bubbles in water, the stability of the wet laser cladding process was poor, and the quality of the cladding layer was low; in addition, the water depth of the laser cladding was small. At present, research on underwater wet laser cladding technology is sparse and mainly focused on theoretical research but has not been reported in the field of engineering applications. The research and application of underwater laser cladding technology mainly focus on the local dry underwater method.

In terms of underwater local dry laser cladding technology, Wang et al. [16,17] studied the underwater powder-feed laser cladding (UPLC) of a TC4 titanium alloy and revealed the evolution mechanism of microstructure, which was obviously different from the in-air powder-feed laser cladding process. In the process of UPLC, the hydrogen content was well controlled and the formation of a hydrogen-induced crack was effectively prevented. The mechanical properties of the underwater laser cladding sample were the same or even better than those of the in-air laser cladding sample. At the same time, they also carried out UPLC of HSLA-100 steel, showing that the cooling rate was significantly increased in the water environment, and the peak temperature reduced; in addition, the hardness of the underwater cladding sample was higher than that of the in-air cladding sample. Compared with underwater wire-feed laser cladding (UWLC) technology, UPLC technology was more susceptible to the influence of the water environment and water pressure. Fu et al. [18,19] prepared a 304 stainless-steel laser cladding layer in situ by UWLC technology and studied the morphology, microstructure and properties of the cladding layer. It was found that the microstructure near the fusion line and in the central region was columnar dendrites and equiaxed grains, respectively. Compared with the in-air laser cladding layer, the microstructure in the underwater laser cladding layer was finer. In addition, due to the action of aerosols, the instability of the underwater laser cladding process would result in a wettability decrease. They also studied the mechanism of the interaction between water, laser and substrate in the underwater laser cladding process and prepared an excellent TC4 single-channel laser cladding layer at an optimized flow rate of protective gas.

At present, research on underwater materials mainly focuses on stainless steel and titanium alloy, while research on aluminum alloy is sparser. Aluminum alloy has been widely used in nuclear power-plant, ocean and other underwater engineering fields due to its high specific strength and strong corrosion resistance [20,21,22,23,24]. In this paper, the influence of the water environment on the quality of multi-channel wire-feed laser cladding was investigated by analyzing the laser cladding process, cladding appearance, geometry characteristics, microstructure, regional segregation and microhardness.

## 2. Experimental Procedure

**Figure 1:**
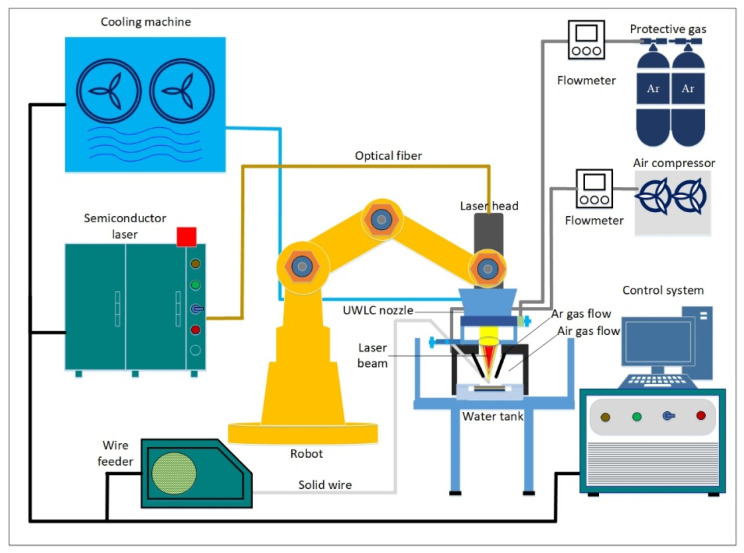
Schematic diagram of the UWLC experiment system.

As shown in Figure 1, the UWLC experiment system was mainly composed of a semiconductor laser, a six-axis robot, a laser cladding nozzle, a wire feeding system and a gas delivery system. The maximum output power of the laser was 5 kW, the wavelength was 915 nm, and the core diameter of the fiber was 800 µm. Laser cladding nozzle was divided into the inner layer and outer layer. In the outer layer, compressed air was passed into the outer layer to discharge water to form a local dry space, and in the inner layer, argon was passed into the inner layer to discharge air to create a protective atmosphere. In order to make the laser cladding process more stable, the front-feed wire method was adopted in this paper. Compared with the post-feed wire and the side-feed wire method, the front-feed wire method had better adaptability to the fluctuation of the metal wire [22,23]. The wire feeding angle was 30°, which could ensure the wire and workpiece absorbed enough energy at the same time. When the distance between the light and wire was set to 0, the melted metal wire could enter the molten pool in the way of liquid-bridge transition, which could reduce the influence of wire fluctuations on cladding stability.

In this test, 5052 aluminum-alloy tube with an outer diameter of 8 mm and wall thickness of 2 mm was used as the base material, and 5052 aluminum-alloy wire with the diameter of 1 mm was used as the filling material. The corresponding chemical composition data from manufacturer are shown in Table 1. The experimental parameters are presented in Table 2.

Before the experiment, aluminum tubes were sanded with sandpaper to remove surface oxides and then cleaned with anhydrous ethanol [24]. The influence of the water environment on the laser cladding process, cladding appearance, geometry characteristics, microstructure, regional segregation and the microhardness of the multi-channel laser cladding samples was studied. After the experiment, the samples were cut along the direction perpendicular to the cladding layers by using line cutting machine and then embedded. The embedded samples were successively polished with sandpaper of different specifications and then polished with polishing agent until the surface of samples had no scratches. Finally, the samples were corroded with self-made etchant liquid (3 mL H_2_SO_4_ + 5 mL HNO_3_ + 3 mL HCl + 2.5 mL HF + 95 mL H_2_O). The cross sections and microstructure of the samples were photographed and analyzed with optical digital microscope. The grain morphology and size of the cladding samples were studied by electron backscatter diffraction (EBSD) under the condition of 10 kV acceleration voltage and 0.2 µm step size. The metal composition of underwater and in-air samples was studied by using electron probe microanalysis (EPMA). The microhardness of the underwater and in-air samples was measured by a Vickers microhardness indentation machine (HV-1000 T) with a loading time of 10 s under a test load of 0.49 N.

## 3. Results and Discussion

### 3.1. Effect of Water Environment on Laser Cladding Process

**Figure 2:**
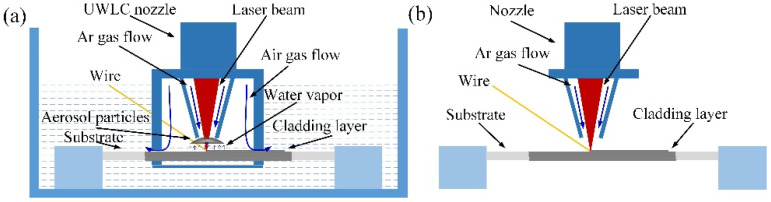
The schematic diagram of underwater and in-air laser cladding processes: (**a**) the underwater laser cladding process; (**b**) the in-air laser cladding process.

The schematic diagram of underwater and in-air laser cladding processes is shown in Figure 2. During the underwater laser cladding process, although the water in the deposition position was displaced, there was still a layer of water film on the surface of the substrate, and therefore, the influence of the water environment on the laser cladding process could not be eliminated. When the laser beam irradiated the water on the surface of the substrate, the water absorbed heat, and it reached the boiling point to form water vapor. In addition, as the temperature of the substrate increased, the water around the substrate also absorbed heat to form water vapor. At the same time, metal burning formed soot in the cladding process. The water vapor and soot gathered in the path of the beam, forming aerosol particles. They had absorption and scattering effects on the transmission of the laser beam, resulting in a decrease in laser energy. According to the report of Guo et al. [25], laser density *I* at laser transmission distance z could be calculated by Formula (1):

(1) $$I\left(z\right)=I_{0}e^{-z\pi \int_{r_{1}}^{r_{2}}Q_{\varepsilon }n\left(r\right)r^{2}dr}$$

where *I*_0_ is the primary laser intensity, *r* is the radius of aerosol particles, *n*(*r*) is the density of aerosol particles, and *Q_e_* is the extinction coefficient of a single spherical aerosol particle. It could be seen that laser intensity is inversely proportional to the density of aerosol particles.

For the underwater laser cladding process, although the passage of protective gas was beneficial to blowing aerosol particles, there was still a small amount of aerosol particles in the beam path due to the binding of the UWLC nozzle. The laser energy absorbed by the base material (BM) and the cladding metal decreased, which reduced the peak temperature in the laser cladding process. In addition, the cooling rate of the laser cladding process was accelerated due to the water environment. For the in-air laser cladding process, there was no water vapor generated and the soot was blown away by the protective gas; thus there was almost no accumulation of aerosol particles in the beam path. Compared with the underwater laser cladding process, the laser energy was absorbed by the BM and the cladding metal increased in the in-air laser cladding process, which resulted in the increase in peak temperature. In addition, because the substrate was surrounded by gas, the cooling rate of the laser cladding process was slowed down.

### 3.2. Cladding Appearance and Geometry Characteristics

**Figure 3:**
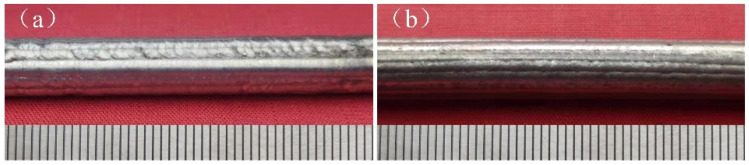
The cladding appearance images: (**a**) the underwater cladding layer; (**b**) the in-air cladding layer.

**Figure 4:**
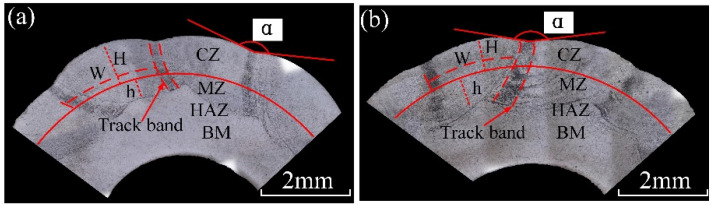
The cross-sectional images: (**a**) the underwater cladding layer; (**b**) the in-air cladding layer.

The underwater and in-air cladding appearances of multiple-track cladding layers are shown in Figure 3. The uniformity of underwater cladding layers was poor, and the surfaces were rough, while the surfaces of in-air cladding layers were smoother, and the uniformity was better. The underwater and in-air transverse sections of multiple-track cladding layers are shown in Figure 4. For both samples, excellent metallurgical bonding was obtained between the multiple-track cladding layers and base metal, and no cracks and pores were observed under the optical microscope, indicating that reliable multiple-track cladding layers were formed on the base metal in the underwater environment. Both in the underwater and in-air multiple-track cladding layers, the cladding regions were uneven, and gaps and track bands between the adjacent cladding tracks were found. However, compared to the in-air multiple-track cladding layers, the underwater multiple-track cladding layers were more uneven, and the gaps and track bands were more obvious. During the multi-channel laser cladding process, the filler wire and base metal were melted or the cladding layers were remelted under the action of the laser beam. Therefore, the multi-channel laser cladding layers could be divided into five regions based on the thermal cycle in the process of multi-channel laser cladding and different material sources [26]. They were, respectively: the cladding zone (CZ), which was areas formed by the melting metal wire deposited on the base metal or other cladding layers; the melting zone (MZ), referring to the melting metal of the substrate; the heat-affected zone (HAZ), which was influenced by the molten pool; the base metal (BM); and the overlapping zone (OZ), referring to the cross areas of adjacent cladding layers. To further describe the geometry of the cross section, the thickness of the CZ (*H*, μm), the depth of the MZ (*h*, μm), the width of the CZ (*W*, μm), the width of the track band (w, μm) and the angle between adjacent CZs (α, °) were measured, and the dilution rate (*D*) and the shape factor (*S*) were evaluated [15]:

(2) D=h/(H+h)

(3) S=W/h

**Figure 5:**
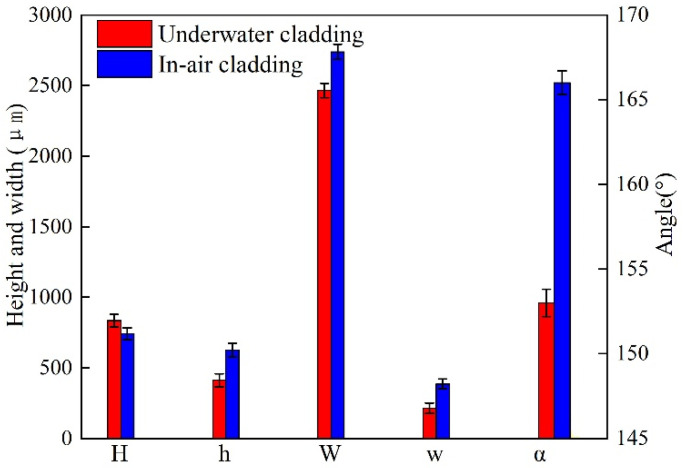
The cross-sectional dimensions of the in-air and underwater cladding tracks.

Figure 5 presents the cross-sectional geometry characteristics of in-air and underwater multiple-track cladding layers. It should be pointed out that the geometry characteristics were the average value of different cladding tracks. As the laser cladding process changed from in-air to underwater environment, the average H decreased from 741 μm to 834 μm, and the average h decreased from 625 μm to 412 μm; in addition, based on Formula (2), D decreased from 0.46 to 0.33. The results show that the peak temperature in the underwater multiple-track laser cladding process was lower than that of the in-air multiple-track cladding process under the same process parameters. The average W of in-air and underwater multiple-track cladding layers was 2739 μm and 2465 μm, respectively. It could be calculated by Formula (3) that the S increased from 4.38 to 5.98, which indicated that the forming quality of the underwater laser cladding sample was worse than that of the in-air laser cladding sample. For in-air cladding layers, the average w and α were 385 μm and 166°, respectively, while average w and α decreased to 214 μm and 153° in underwater cladding layers. The results show that the surface of the underwater laser cladding sample was rougher than that of the in-air laser cladding sample. By comparing the geometry characteristics of in-air and underwater multiple-track cladding layers, it was further verified that the existence of the water environment could lead to a decrease in laser power density and the instability of the cladding process, and the cooling effect of the water environment increased the cooling rate of the deposited metal and reduced the high-temperature residence time.

### 3.3. Microstructure

**Figure 6:**
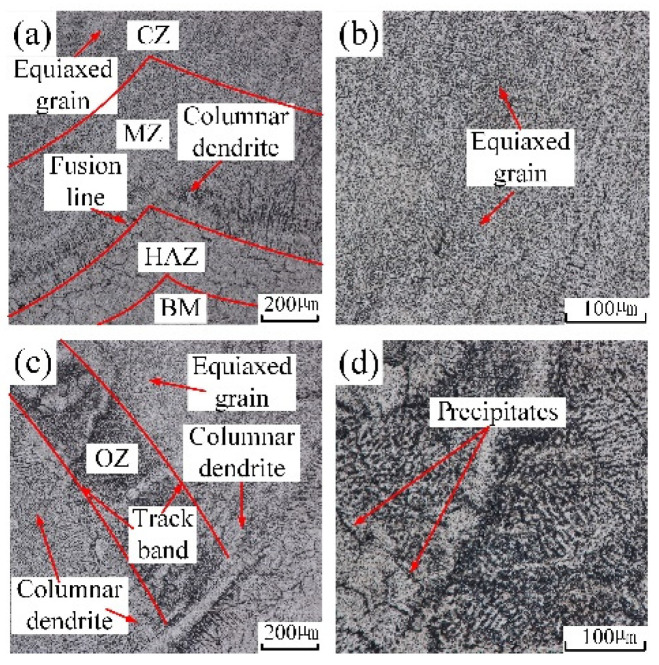
The microstructure in distinctive zones of in-air cladding layer: (**a**) the microstructure near fusion line; (**b**) the local amplification of CZ; (**c**) OZ; (**d**) the local amplification of OZ.

**Figure 7:**
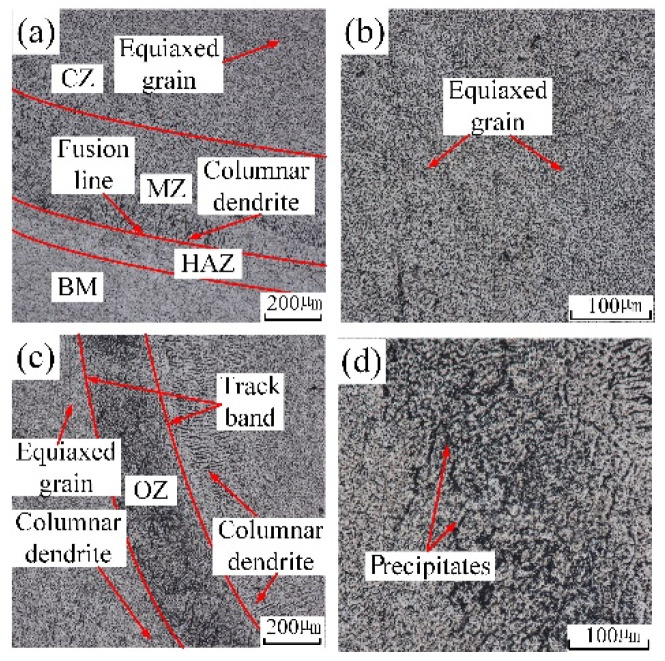
The microstructure in distinctive zones of underwater cladding layer: (**a**) the microstructure near fusion line; (**b**) the local amplification of CZ; (**c**) OZ; (**d**) the local amplification of OZ.

Figure 4a,b show the cross-section macrostructure of in-air and underwater multiple-track cladding layers, and the microstructure of in-air and underwater samples extracted from different areas and captured by the optical microscope is displayed in Figure 6 and Figure 7. When the laser was used to irradiate the surface of the substrate and wire, a molten pool formed in the substrate and solidified after the laser beam was removed. During the solidification process of the molten pool, the grain growth direction mainly depended on the temperature gradient (G), while the grain morphology was determined by the temperature gradient (G) and the grain growth rate (R) [27]. Figure 6a shows the microstructure near the fusion line of the in-air multiple-track laser cladding sample. One side of the fusion line was the HAZ, and the other side was the MZ. Due to the effect of the high temperature of the molten pool, the grains near the fusion line grew, and thus the microstructure in the HAZ was relatively coarse equiaxed grains. The rapid loss of heat through the substrate at the edge of the molten pool resulted in a large G and a small R, thus forming columnar dendrites in the MZ. Since the direction of the temperature gradient was perpendicular to the center of the molten pool from the fusion line, the columnar dendrites grew almost perpendicular to the fusion line toward the center of the molten pool [15]. Figure 6b shows the microstructure of the CZ, which was mainly composed of equiaxed grains. Because the heat dissipation in the CZ was mainly carried out by air and the solidified MZ, the cooling rate reduced and the G was suppressed. Figure 6c shows the microstructure in the OZ of adjacent cladding layers. In the middle of the track bands, there were relatively thick equiaxed grains and columnar dendrites, and precipitates were generated on its surface. On one side of the track bands were columnar dendrites growing almost perpendicular to the track line, and on the other side of the track bands were equiaxed grains and columnar dendrites. Figure 6d shows the local amplification of the middle region of the track bands in Figure 6c. There were two kinds of precipitated phases: grain boundary precipitates and matrix precipitates. They presented dense and point-like distribution on the surface of grains.

The grains’ distribution of the underwater multiple-track laser cladding layers in each region was basically the same as that of the in-air multiple-track laser cladding layers. By comparing Figure 6a,c with Figure 7a,c, it could be found that the width of the underwater HAZ, the height of the MZ and the width of the OZ were smaller. Due to the fast cooling rate of the water environment, the laser energy conduction on the cladding sample was accelerated, and the sample experienced a short thermal cycle time. The width of the underwater HAZ, the height of the MZ and the width of the OZ decreased. In addition, the precipitated phases in the underwater OZ reduced. By comparing Figure 6d with Figure 7d, it could be found that the distribution of underwater precipitated phases was more uniform. During the process of underwater laser cladding, due to the rapid cooling rate of the water environment, the precipitation of the precipitated phase was inhibited, and the parent-phase solid solution was significantly supersaturated. The sample was kept at room temperature for a period of time, and solute atoms had a certain diffusion ability; thus partially supersaturated solid solution would still have the second-phase precipitation reaction, forming precipitated phases. However, in the in-air laser cladding process, the precipitation of the precipitated phase was not inhibited due to the relatively low cooling rate. Therefore, compared with the in-air cladding sample, the precipitated phases in the OZ of the underwater cladding sample reduced.

**Figure 8:**
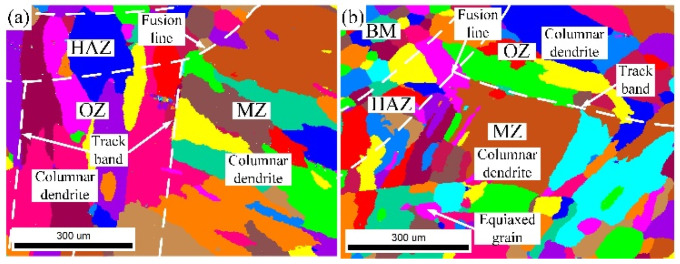
The grain morphology near the in-air and underwater fusion line: (**a**) the in-air grain morphology; (**b**) the underwater grain morphology.

**Figure 9:**
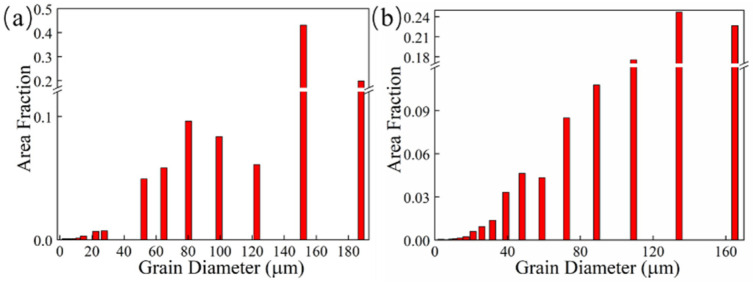
The distribution of grain sizes in the in-air and underwater OZs: (**a**) the in-air OZ; (**b**) the underwater OZ.

**Figure 10:**
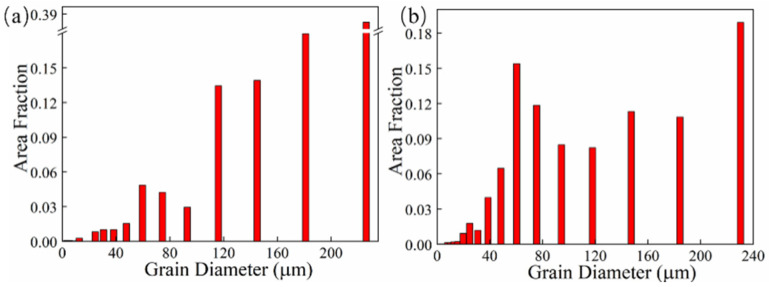
The distribution of grain sizes in the in-air and underwater MZs: (**a**) the in-air MZ; (**b**) the underwater MZ.

The grain morphology near the in-air and underwater fusion line is shown in Figure 8. Part of the grains in the HAZ grew through the fusion line to the MZ, which indicated that there was an obvious co-crystalline relationship between the HAZ and MZ. The grain was ununiform, and some small grains formed inside the large grains in both the in-air and underwater MZs. The distribution of grain sizes in the in-air and underwater OZs and MZs is shown in Figure 9 and Figure 10. The maximum grain size in the in-air OZ and MZ was 187 µm and 226 µm, and the average grain size of the OZ and MZ was 139 µm and 168 µm, while the maximum grain size in the underwater OZ and MZ was 165 µm and 230 µm, and the average grain size of the OZ and MZ was 118 µm and 128 µm. The cool rate of the water environment was faster than that of the air; in addition, the underwater peak temperature was lower than that of in-air, and therefore, the underwater grain size was smaller.

### 3.4. Regional Segregation

**Figure 11:**
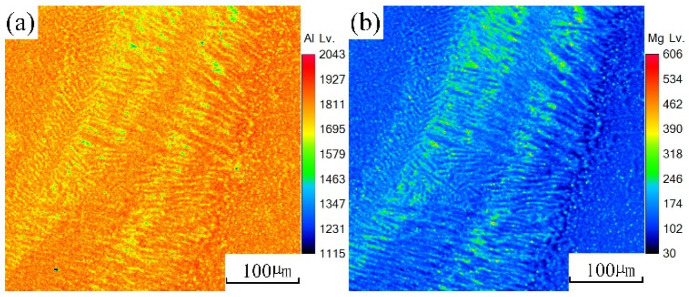
The sweep diagrams of different elements in the in-air cladding layer: (**a**) Al; (**b**) Mg.

**Figure 12:**
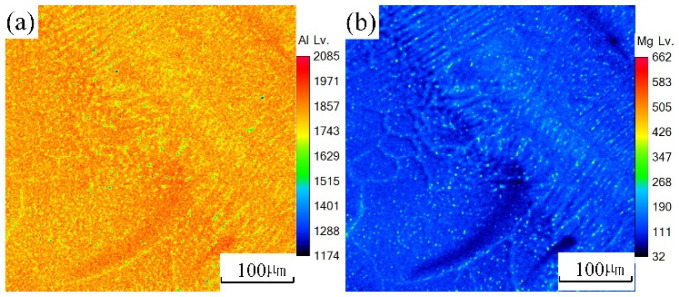
The sweep diagrams of different elements in the underwater cladding layer: (**a**) Al; (**b**) Mg.

An EPMA test was carried out on the underwater and in-air laser cladding samples, and the sweep diagrams are shown in Figure 11 and Figure 12, respectively. It could be found that the content of magnesium element in the in-air laser cladding sample increased gradually from the beginning to the end of columnar dendrite growth, while the content of aluminum element decreased gradually. Significant positive segregation occurred in the columnar dendrite region growing outward perpendicular to the OZ. However, only a small amount of magnesium element was enriched in the interdendrite, indicating that the positive segregation was not significant in the underwater laser cladding sample.

Definition of equilibrium partition coefficient k: the ratio of solute concentrations in two equilibrium phases of solid and liquid at a certain temperature. The equilibrium distribution coefficient k was less than 1. According to the distribution law of solute atoms, during the unbalanced crystallization process, solute atoms did not diffuse in the solid phase, and thus the concentration of solute atoms in the solid phase solidified first was lower than the average composition. In the process of in-air laser cladding, the crystallization rate was slow and the atomic diffusion in the liquid was sufficient. The solute atoms could diffuse to the distant crystallization front through convection, and thus the concentration of the post-crystallization liquid gradually increased. After the crystallization, the concentration of solute atoms at the beginning and the end of the columnar dendrites varied greatly. That is, the positive segregation was significant. During the process of underwater laser cladding, the crystallization rate was fast, there was almost no convection in the liquid phase, the atomic diffusion was insufficient, and thus the solute atoms were only enriched in the interdendrite, and the positive segregation was not significant.

### 3.5. Microhardness

**Figure 13:**
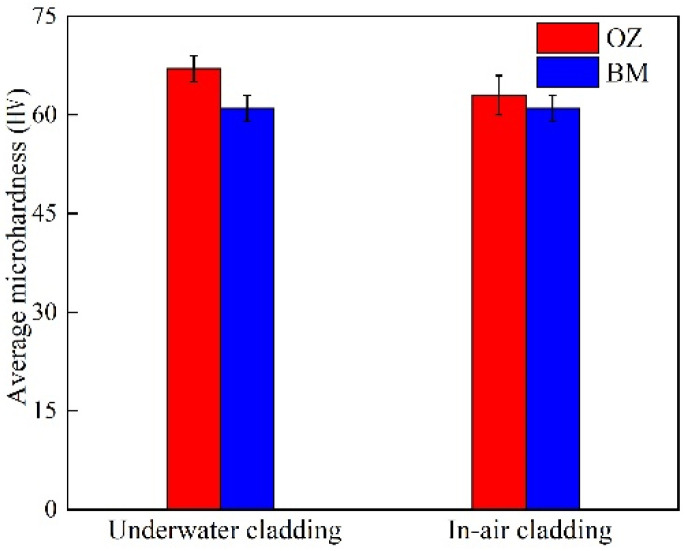
The microhardness distribution in different zones of underwater and in-air cladding layers.

The average microhardness values of the underwater and in-air OZ are shown in Figure 13. The average microhardness value of the underwater OZ was 67 HV, while the average microhardness value of the in-air OZ was 63 HV. It could be found that the underwater average microhardness value was higher than that of in-air. On the one hand, increasing temperature led to the formation of more precipitates at the matrix and grain boundaries, which would increase the microhardness of material. On the other hand, the increase in temperature also made the grain grow. According to the Hall–Petch theorem, the average microhardness value would decrease. It could be seen from the results that the combined actions made the in-air microhardness of material decrease.

## 4. Conclusions

In order to study the effect of the water environment on wire-feed laser cladding of the 5052 aluminum alloy, we compared the quality of in-air and underwater laser cladding under the same process parameters, and the following conclusions were drawn:

(1) The uniformity of in-air cladding layers was better than that of underwater. For both samples, excellent metallurgical bonding was obtained between the multiple-track cladding layers and base metal. As the laser cladding process changed from in-air to underwater environment, the D, w and α decreased, but the S increased.
(2) For the in-air and underwater samples, the microstructure of the MZ and CZ was all columnar dendrites and equiaxial dendrites, respectively. In addition, the microstructure of the OZ was all composed of columnar dendrites and equiaxial dendrites. On one side of the track bands were columnar dendrites growing almost perpendicular to the track line, and on the other side of the track bands were equiaxed grains and columnar dendrites.
(3) Significant positive segregation occurred in the columnar dendrite region of the in-air cladding sample growing outward perpendicular to the OZ. However, only a small amount of magnesium element was enriched in the interdendrite of the underwater cladding sample. It indicated that the water environment was helpful to reduce the positive segregation in the columnar dendrite region.
(4) There were two kinds of precipitated phases: grain boundary precipitates and matrix precipitates. They presented dense and point-like distribution on the surface of grains. Compared with the in-air cladding sample, the precipitated phases in the OZ of the underwater cladding sample reduced. The average microhardness value of the underwater OZ was higher than that of the in-air OZ.

## Figures and Tables

**Table 1:** Chemical compositions of the 5052 aluminum alloy (mass fraction/%).

Si	Fe	Cu	Mn	Mg	Cr	Ti	Zn	Al
≤0.25	≤0.4	0.1	0.1	2.2–2.8	0.15–0.35	—	0.1	Bal.

**Table 2:** Process parameters of comparative experiment between UWLD and IWLD.

Process Parameters	Depth of Water (mm)	Heat Input (kJ/mm)	Wire Feeding Speed (cm/min)	Gas Flow Rate (L/min Ar)	Rotation Angle (°)
Value	0.20	0.39	110	35	20

## Data Availability

Not applicable.
